# Novel Endoscopic Management of Eroding Laparoscopic Adjustable Gastric Band: A Case Series

**DOI:** 10.1155/2018/2747852

**Published:** 2018-12-30

**Authors:** Dionysios Dellaportas, Constantinos Nastos, Theodosios Theodosopoulos, George Fragulidis, Andreas Polydorou, Antonios Vezakis

**Affiliations:** 2 Department of Surgery, ‘Aretaieion' University Hospital, Medical School of Athens, Greece

## Abstract

Complications of laparoscopic adjustable gastric bands include migration and slippage of the band, dilation of the proximal gastric pouch, troublesome gastroesophageal reflux symptoms, and erosion of the stomach. The latter occurs in 0.6-12.7% of cases and necessitates removal of the band. Several open and laparoscopic surgical techniques have been described for band extraction, while fully endoscopic techniques have emerged and proven safe. Three cases of eroding gastric bands treated in a single center with fully endoscopic removal of the band are analyzed in this study. Novel use of the duodenoscope and endoscopic retrograde cholangiopancreatography instruments and accessories is described, in order to perform endoscopic division of the plastic band and retraction through the mouth. All three cases were successfully treated utilizing this novel technique. Gastric wall erosion from the band has nonspecific symptoms and various predisposing factors. Removal of the foreign material is required. Endoscopic procedures are effective in 77-92% of cases, avoiding general anaesthesia with low surgical morbidity. As a result patients are discharged early resolving quicker to a normal diet.

## 1. Introduction

Laparoscopic adjustable gastric banding (LAGB) is a common restrictive bariatric procedure [1]. It has well recognized benefits as ease of insertion, adjustable effectiveness, and reversibility. On the other hand migration and slippage of the band, dilation of the proximal gastric pouch, gastroesophageal reflux symptoms, and erosion of the stomach at the site of the band are complications constituting significant morbidity of LAGB [2]. The latter can cause dysphagia, severe reflux symptoms, infective complications, and perforation of the stomach, necessitating removal of the band [3].

In cases where a large portion of the band has eroded and migrated into the stomach lumen, an endoscopic approach to remove the band is utilized, avoiding laparoscopic or open surgery [4]. A small case series of a single center experience is reviewed herein, describing the endoscopic technique using conventional low-cost endoscopic equipment.

## 2. Case Series

### 2.1. Case 1

**Figure 1:**
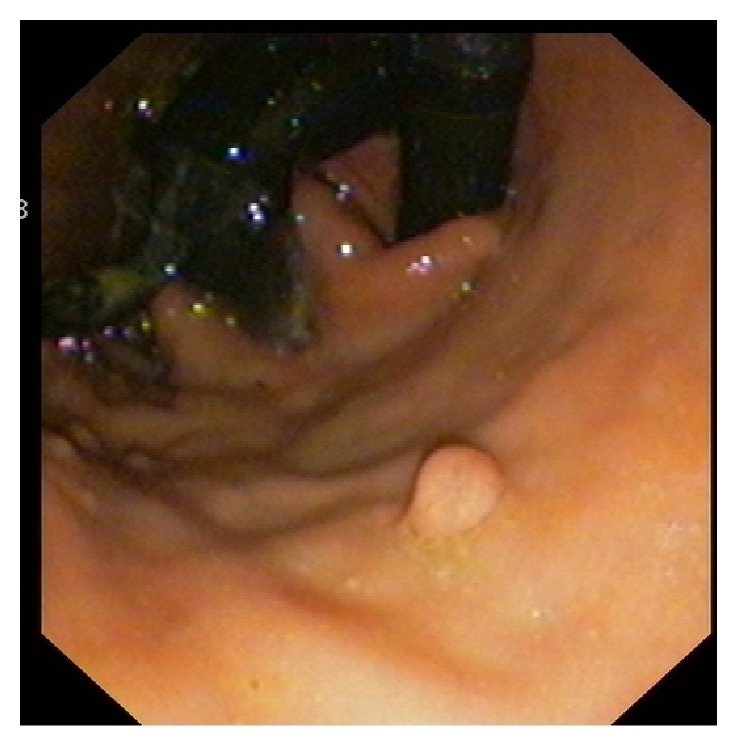
Endoscopic view on retroflexion of eroding band with locking mechanism.

A 47-year-old woman underwent LAGB in 2005 with a BMI of 41 and after significant weight loss the band was fully deflated in 2010, however kept in situ. The patient developed severe gastroesophageal reflux disease (GERD) symptoms a year later and was treated with omeprazole 20mg twice daily. The patient refused upper gastrointestinal endoscopy until she developed dysphagia and signs of infection of the port site over the past few months. On endoscopy she was diagnosed with a migrated band which has eroded into her stomach in more than 50% of its circumference (Figure 1). This finding was confirmed on a computed tomography (CT) scan excluding any other pathology.

Endoscopic removal of the band was decided and performed using a conventional JAG wire (Boston Scientific Corporation) and the mechanical emergency lithotripter handle (Olympus).

### 2.2. Technique

**Figure 2:**
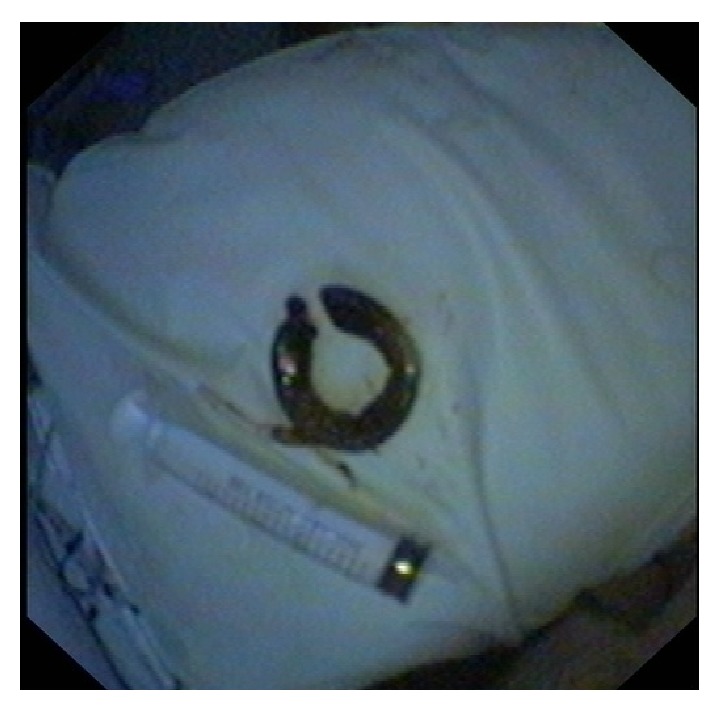
Adjustable gastric band after removal.

Firstly, the band port was removed under local anaesthetic with the patient in supine position. Next, the patient was rolled into classic esophagogastroduodenoscopy (EGD) left decubitus position and propofol sedation was administered. EGD was performed using the duodenoscope which confirmed the eroding migrated gastric band. A JAG wire was advanced through the endoscope and passed around the lumen-protruding band. To complete the above maneuver a sphincterotome was utilized to achieve the steep ankle needed for placing the wire around the foreign material. The authors view is that the latter endoscopic maneuver is easily feasible using the side-viewing duodenoscope instead of the forward viewing gastroscope. Both JAG wire ends were placed through the mechanical biliary lithotripter parts (narrow metal tube and tourniquet) and the endoscope was placed once more in position checking the cutting wire mechanism, making sure no mucosa is entrapped into the JAG wire loop. Under direct endoscopic vision the band was divided easily as the JAG wire was squeezed and worked as a cutting device. Afterwards the lithotripter system and wire was withdrawn and a polypectomy snare was used to grasp the divided band end closest to the locking band mechanism and pulled around in order to bring into the stomach lumen the whole band circumference. Finally, with the band grasped with the snare and with careful traction the eroding foreign material was extracted through the mouth (Figure 2). A final endoscopy confirmed that there was no bleeding from mucosal injury or a visible gastric defect.

The patient was started on clear fluids two hours later and was discharged on the first postoperative day. Her symptoms of dysphagia fully resolved and her reflux symptoms have substantially improved.

### 2.3. Case 2

**Figure 3:**
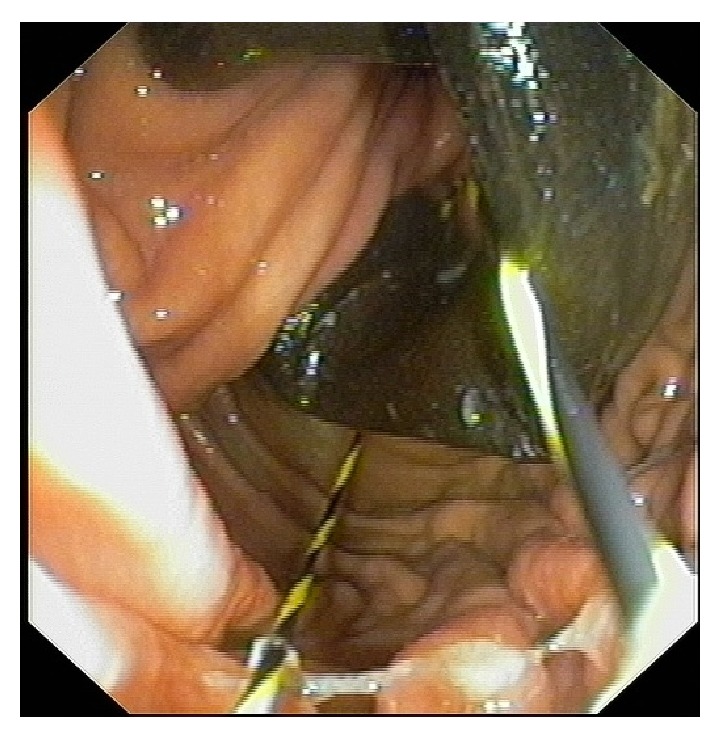
Endoscopic view of JAG wire passed around the eroding band.

A 56-year-old woman who had LAGB six years earlier was investigated for epigastric discomfort. On EGD it was demonstrated that the gastric band has eroded into the gastric lumen (Figure 3). The band was fully deflated and the patient was scheduled for therapeutic endoscopy and gastric band removal. Using the technique described above the eroding band was extracted successfully. The patient had an uneventful course and was discharged on the first postoperative day. Her symptoms resolved as well; however, the patient regained weight and was referred to a bariatric surgeon for further consultation.

### 2.4. Case 3

**Figure 4:**
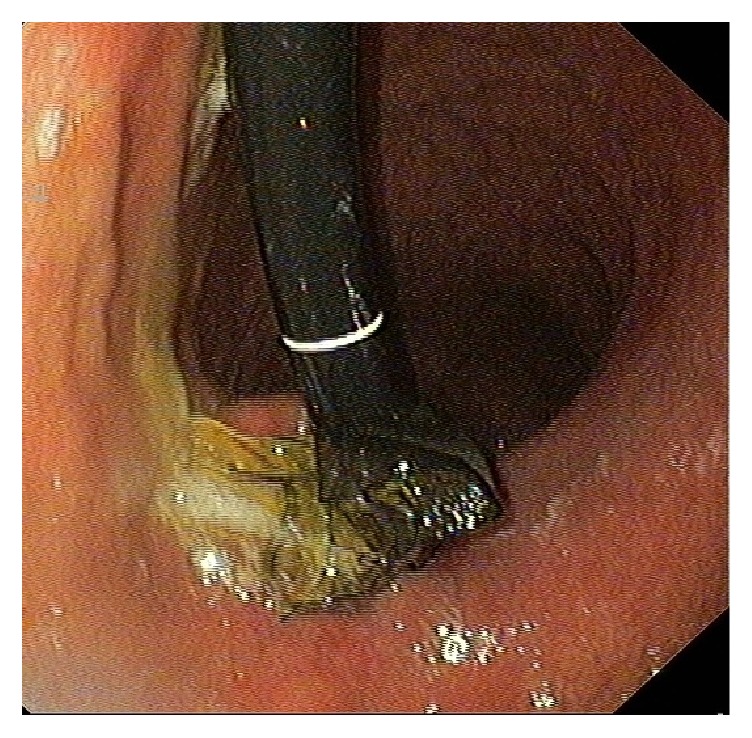
Endoscopic view of eroding band with locking mechanism.

A 21-year-old female was admitted to the hospital with postprandial vomiting and epigastric pain. She has undergone LAGB three years ago with a BMI of 43 and at the time she has lost 30% of her excess body weight. On admission the band was fully deflated; however, her symptoms persisted. On abdominal X-ray the band's phi ankle was 0' and proximally migrated. EGD was performed and showed gastric band erosion in about 30% of its waist (Figure 4). On the following day the patient underwent a successful endoscopic removal of the eroding gastric band, using the above described technique.

After a normal water soluble swallow test on postoperative day 1, her diet was advanced and discharged on the second postoperative day. The water soluble swallow test was performed because the patient complained of epigastric pain and discomfort, without being an absolute requirement for diet build up.

## 3. Discussion

LAGB is a popular bariatric procedure achieving significant weight loss with low morbidity rate [5]. Band erosion into the gastric lumen is a long-term complication well described in several studies occurring in 0.6-12.7% [6–8]. Patients symptoms are nonspecific as epigastric discomfort or pain, exacerbation of reflux-like symptoms, dysphagia, and port site infection.

It is suggested that inadvertent intraoperative gastric wall injury, overfilling of the band, and surgeon's inexperience are the main factors contributing to the occurrence of this serious complication [9]. Filling volumes of 10-12ml are considered high and when used for long duration predispose to stomach erosion [10]. In this case series all gastric bands were of the adjustable Lap-Band 9.75cm type. It is stated that the need for band overfilling might be an indication of band erosion, while the Vanguard Lap-Band and revision of bariatric surgery with the use of gastric bands have higher rates of erosive complications [9]. Interestingly, port site infection as in the first case presented herein is considered an early sign of gastric erosion [11].

Despite agreement that this complication should be treated with gastric band removal, there is lack of consensus as to the best approach of removing the eroding foreign material. Laparoscopic removal is the most utilized option, in which anterior gastrotomy and removal of the band are performed; however, endoscopic techniques have emerged and reported to be very effective avoiding general anaesthesia and operative risks [12]. In a large series, Neto et al. report an 85% successful endoscopic removal with 5.8% morbidity, and another interesting study reports 92% successful extraction with endoscopy only [13]. Both studies emphasize the low morbidity constituting mostly in pneumoperitoneum, which resolved without intervention, and bleeding from gastric mucosal injury. It is suggested that when 50% of the band circumference is endoscopically visible into the gastric lumen with the locking mechanism, then endoscopic removal is feasible [14]. Otherwise, laparoscopic or hybrid surgical and endoscopic options are safer. If the locking mechanism has not eroded into the stomach, then pulling it endoscopically might cause gastric wall injury since this is the thickest part of the band [15].

The endoscopic technique described above mainly differs in that the duodenoscope is used for endoscopic view of the eroding band. It provides greater ankle range and maneuvering options in the endoscopist's efforts to place the cutting wire around the band. Without any extra cost, conventional endoscopic retrograde cholangiopancreatography (ERCP) equipment, and in more detail, a conventional sphincterotome, a JAG wire, and with the off-label use of the biliary mechanical lithotripter, the band can be cut successfully. Finally, for retraction a polypectomy snare or a stent grasper is the fourth endoscopic instrument required to complete the procedure.

For the classic endoscopic procedure, after the band port removal under local anaesthetic, the forward viewing gastroscope is used. The Gastric Band Cutter is used and a stainless steel wire is advanced through the gastroscope working channel into the stomach and past the pylorus, to be easier to retrieve later on. The gastroscope is fully retrieved, while the wire is left in situ. The endoscope is introduced once more and the endoscopist is trying to either manipulate the wire and place it around the eroding band, which may be a very difficult maneuver to achieve, or passes the endoscope itself between the band and the stomach wall, in order to retrieve the wire and while pulling the endoscope back into the esophagus having the cutting wire looped around the eroding band. The latter maneuver may also be a cumbersome endoscopic task. In the technique described above with the duodenoscope, wire manipulation becomes easier through the sphincterotome, which is already half-looped. Both wire ends are placed into an external metal tube and through the tourniquet of the Gastric Band Cutter. The rest of the procedure is similar to the one described above [8].

In conclusion, endoscopic removal of eroding gastric bands is an effective option managing this troublesome complication. It allows avoidance of postoperative morbidity and achieves early discharge of patients, with the extra benefit of minimal cost.
